# Effect of psychotherapy on peripheral BDNF concentration levels in patients with bipolar disorder. A systematic review

**DOI:** 10.1192/j.eurpsy.2023.1477

**Published:** 2023-07-19

**Authors:** M. Valtueña García, L. Rubio Rodríguez, L. Sánchez-Pastor, I. Martínez-Gras, R. Rodríguez-Jiménez

**Affiliations:** 1 Psiquiatria, Instituto de Investigación Sanitaria Hospital Universitario 12 de Octubre (imas12), Madrid; 2 Psiquiatria, (3) Unidad de Hospitalización psiquiátrica. Fundación Hospital de Jove. Gijón, Spain

## Abstract

**Introduction:**

Psychotherapy is a treatment of proven efficacy in bipolar disorder (BD), but little is known about the molecular and cellular mechanisms that it produces in the brain. Brain-derived neurotrophic factor (BDNF) is thought to be important in neuroplasticity and could be increased by psychopharmaceuticals and psychotherapy in BD patients, but evidence in the literature is limited.

**Objectives:**

To analyze the scientific studies that relate psychotherapies with the increase in BNDF levels in patients with BD.

**Methods:**

Systematic review with PRISMA recommendations in PUBMED and Web of Science in July 2022. The search was performed using the combination of keywords “bipolar disorder” AND (“BDNF” OR “Brain Derived Neurotrophic Factor”) AND “psychotherapy”.

**Results:**

With the initial search, 839 studies were obtained, finally 8 articles were analyzed. The available literature supports the role of psychotherapy in increasing BNDF in patients with BD.

**Conclusions:**

BDNF could be a biomarker of therapeutic efficacy in BD. Psychotherapy increases BDNF levels. No differences were found between the different types of psychotherapies. More studies are needed to determine the mechanisms by which psychotherapies produce molecular changes in the brain.

**Disclosure of Interest:**

None Declared

